# Editorial: Podocyte Pathology and Nephropathy

**DOI:** 10.3389/fendo.2015.00145

**Published:** 2015-09-17

**Authors:** Barbara Lewko, Gavin Iain Welsh, Maciej Jankowski

**Affiliations:** ^1^Department of Pathophysiology, Faculty of Pharmacy, Medical University of Gdansk, Gdansk, Poland; ^2^Laboratory of Molecular and Cellular Nephrology Gdansk, Miroslaw Mossakowski Medical Research Center of the Polish Academy of Sciences, Warsaw, Poland; ^3^Academic Renal Unit, University of Bristol, Bristol, UK; ^4^Department of Clinical Chemistry, Medical University of Gdansk, Gdansk, Poland

**Keywords:** podocytes, diabetes, kidney disease, proteinuria, foot processes, exosomes

Podocytes, glomerular visceral cells of epithelial origin, are the direct target of both immune and non-immune forms of injury in many diverse glomerular diseases. They do not typically proliferate in the mature kidney, which means that damaged cells are not replaced by new ones. It is now generally accepted that dysfunction or loss of these cells underlies progression of almost all glomerulopathies. Progressing glomerular diseases, of which diabetic nephropathy (DN) is the most common, are the most frequent cause of end-stage renal disease. Therefore, identification of the pathways that lead to podocyte injury is essential for developing more effective, targeted therapies. The articles presented in this research topic give a comprehensive overview of recent discoveries into the mechanisms of podocyte impairment and discuss the possibilities of podocyte-targeted therapies.

Each podocyte contacts its neighboring cells via a slit diaphragm (SD) connecting their interdigitating foot processes (FPs). Via transmembrane adhesion receptors in the basolateral domain of the FP, the podocytes interact with the glomerular basement membrane (GBM). Both these forms of adhesions are crucial for maintaining podocyte structure, which in turn determines the integrity and permeability of the glomerular filtration barrier. As highlighted by Lennon et al. (1), the cell–cell and cell–matrix junctions drive the coordinated response of podocytes to environmental cues in order to regulate glomerular filtration. Recent findings indicate that there are two distinct FP types, anchoring FPs (AFPs) and ordinary FPs (OFPs), both of which are involved in the regulation of fluid outflow from the subpodocyte space (2). The components of SD and adhesion complexes in FPs transduce signals from outside of the podocyte to the actin cytoskeleton inside the cell. Up to now, almost 100 actin associated proteins have been discovered in mammalian podocytes, with distribution specific for apical, SD, and basal domains of the FP membrane (2). Dysregulation of signaling is likely to lead to actin reorganization and podocyte foot process effacement, which is typically observed in proteinuric diseases. In contrast to traditional interpretations of this loss of shape as a pathological derangement, Kriz et al. (3) indicate that adhesions are reinforced in effaced FPs and therefore it seems to be a protective mechanism against detachment. Alterations in podocyte phenotype and structure are particularly prominent when proteinuria reaches nephrotic range. It is accepted that loss of the specialized podocyte morphology is associated with transition from epithelial to a more mesenchymal phenotype irrespective of the underlying causes that include both genetic defects and mediators from the microenvironment. However, May et al. (4) note that podocytes display partial features of both mesenchymal and epithelial cells. Therefore, dependent on the clinical conditions, dedifferentiation in disease could result in regression to either of these states. Upon treatment, these changes are reversible only if the insult is not very severe, as for example in minimal change disease (MCD). In focal segmental glomerulosclerosis (FSGS), phenotypic dedifferentiation of podocytes is not only irreversible but progressive.

Using a rat model, Kriz et al. (3) have performed a detailed structural study demonstrating how podocytes reinforce attachment to the GBM and how they detach. It appears that if the protective mechanisms fail, viable podocytes, mostly in clusters, detach from the GBM. Some of them may reach the renal pelvis as living cells, while other may develop contacts to the parietal epithelium, forming crescents that connect glomerular capillaries with the Bowman’s capsule. Podocyte depletion represents one of the earliest cellular lesions affecting the diabetic kidney, and decreased number of podocytes in glomeruli is the strongest predictor of progression of both type 1 and 2 DN. Activation of protein kinase C (PKC) seems to play a critical role in pathogenesis of DN. Teng et al. (5) point out that conventional as well as atypical forms of PKC, which play a pivotal role in the regulation of podocyte physiology, may be a destructive factor when hyperactivated in disease conditions. PKC activation results in downregulation of podocyte and SD structural proteins such as P-cadherin or β-catenin, which may contribute to the disruption of podocyte integrity. In DN, the PKC isoforms may also mediate the high glucose-induced overproduction of VEGF and increased TGFβ signaling in podocytes, with subsequent impairment of the glomerular filtration barrier. Recent findings reveal that abnormal intracellular accumulation of sphingolipids modulates podocyte functions in glomerular disorders of both genetic and non-genetic origin. Based on their experimental results, Merscher and Fornoni (6) report that in FSGS, suPAR-dependent αVβ3 integrin activation decreased expression of sphingomyelin-like phosphodiesterase 3b (SMPDL3b) resulting in increased accumulation of sphingomyelin, which is associated with remodeling of the podocyte actin cytoskeleton, loss of stress fibers, and a shift from a migratory to an apoptotic phenotype. In contrast, in the diabetic kidney SMPDL3b expression was elevated, nonetheless rendering podocytes more susceptible to apoptosis. These observations indicate that podocyte responses to sphingolipids are complex and require additional research.

Hereditary, but also sporadic, nephrotic syndrome (NS) is frequently associated with mutations in podocyte genes encoding functional and structural proteins. However, currently known mutations explain <40% of NS cases (7). Moreover, immunosuppression appears to be effective in about 8–10% of genetic disorders. Recent advances in our knowledge about the podocyte transcriptosome and proteasome have led to identification and characterization of novel disease-causing variants and disease-modifying genes. At present, almost 50 podocyte genes directly associated with human NS have been identified. It has also become apparent that monogenic defects correlating with some morphological changes in podocytes do not explain completely the pathogenesis of congenital podocytopathies.

An understanding of the mechanisms of podocyte impairment will allow for the design of targeted therapeutic approaches that may prevent deterioration of glomerular function, e.g., in allograft recipients. The frequency of recurrent as well as newly developed post-transplant kidney diseases, such as DN, is relatively high and now it is clear that podocytes are the initial site of injury. Detailed pathogenesis of post-transplant diabetes is not known; however, numerous risk factors have been identified (8). Although direct treatment of genetic disorders still remains a question for the future, certain biochemical pathways in podocytes already seem to be a promising target for current therapies. For example, it has been shown recently that SMPDL3b in podocyte lipid rafts is a direct target for rituximab, which prevents downregulation of this protein and podocyte injury (7). Furthermore, calcineurin inhibitors (CNIs) that, together with glucocorticoids and mTOR inhibitors, have been traditionally used as anti-inflammatory agents have been shown to also act directly on podocytes (9). Diverse actions of CNIs on podocytes include stabilizing the actin cytoskeleton and inhibiting NFAT-dependent podocyte apoptosis. Other immunosuppressive drugs, such as abatacept or belatacept, also directly target the podocytes, suggesting this is an exciting area for future research (8).

Early, sensitive, and specific diagnosis of ongoing podocyte injury is still lacking. Microalbuminuria, which results from podocyte dysfunction, has been accepted as the earliest marker of DN. However, patients presenting with microalbuminuria already show advanced damage of the glomerular filtration barrier (10), while nephrinuria may precede this manifestation (11). It has been shown recently that urinary podocyte damage biomarkers correlate with cystatin C and eGFR even in normoalbuminuric patients with type 2 diabetes (11). Yet, due to technical problems, such as low quantities of podocyte proteins or their proteolytic digestion, using free urinary podocyte proteins as diagnostic markers appears to be questionable. Musante et al. (12) describe a promising new method in which exosomes carrying specific podocyte biomarkers are isolated from urine. In one pilot study, the levels of urinary exosomal mRNA for cystatin C showed a marked upregulation after the induction of podocyte damage in the puromycin aminonucleoside nephrosis (PAN) rat model (13). However, this was accompanied by the *de novo* expression of cystatin C not only in podocytes but also in tubular epithelial cells. On the other hand, in an experimental model of DN, which is associated with podocyte injury, cystatin C immunoreactivity in tubules was not changed (14). So far, there are only few studies referring to the urinary exosomal mRNA, and data concerning levels and cellular origin of cystatin C mRNA in urine are still missing. Nevertheless, correlation between cystatin C and podocyte-specific genes in urinary exosomes could probably allow for early specific diagnosis of podocyte damage prior to the symptomatic podocytopathy.

## Conflict of Interest Statement

The authors declare that the research was conducted in the absence of any commercial or financial relationships that could be construed as a potential conflict of interest.
